# Biphasic Calcium Phosphate Bioceramics for Orthopaedic Reconstructions: Clinical Outcomes

**DOI:** 10.1155/2011/129727

**Published:** 2011-06-28

**Authors:** Carlos A. Garrido, Sonja E. Lobo, Flávio M. Turíbio, Racquel Z. LeGeros

**Affiliations:** ^1^Department of Orthopaedic Surgery, Hospital São Bento Cardioclinica Ltda., Rua Crucis 50, 30.360-290 Belo Horizonte, Minas Gerais, Brazil; ^2^Department of Orthopedic Surgery, Federal University of São Paulo, Rua Borges Lagoa 783, 04038-032 São Paulo, Brazil; ^3^Department of Morphology, Federal University of São Paulo, Rua Botucatu 740, 04023-900 São Paulo, Brazil; ^4^Department of Biomaterials and Biomimetics, New York University College of Dentistry, 345 East 24th Street, New York, NY 10010, USA

## Abstract

BCP are considered the most promising biomaterials for bone reconstruction. This study aims at analyzing the outcomes of patients who received BCP as bone substitutes in orthopaedic surgeries. Sixty-six patients were categorized according to the etiology and morphology of the bone defects and received scores after clinical and radiographic evaluations. The final results corresponded to the combination of both parameters and varied from 5 (excellent result) to 2 or lower (poor result). Most of the patients who presented cavitary defects or bone losses due to prosthesis placement or revision, osteotomies, or arthrodesis showed good results, and some of them excellent results. However, patients with segmental defects equal or larger than 3 cm in length were classified as moderate results. This study established clinical parameters where the BCP alone can successfully support the osteogenic process and where the association with other tissue engineering strategies may be considered.

## 1. Background

Commercial bioactive ceramics used for bone repair include calcium carbonate (CaCO_3_, in aragonite form), calcium sulfate (CaSO_4_·2H_2_O, plaster of Paris), calcium phosphates, and bioactive glasses. Calcium phosphate ceramics include beta-tricalcium phosphate [*β*-TCP, Ca_3_(PO_4_)_2_], hydroxyapatite [HA, Ca_10_(PO_4_)_6_(OH)_2_], and biphasic calcium phosphate (BCP) (consisting of an intimate mixture of HA and *β*-TCP of varying HA/*β*-TCP ratios).

Unknowingly, the first preclinical application of BCP was reported by Nery et al. in 1975 using a calcium phosphate they described as “tricalcium phosphate” but was analyzed using X-ray diffraction as a mixture of HA and *β*-TCP [1] and, consequently, such a mixture was described as a biphasic calcium phosphate, BCP [2, 3]. The elaboration of BCP was first introduced by LeGeros in 1986 [4]. The efficacy of BCP was based on the preferential dissolution of the *β*-TCP compared to HA, allowing the manipulation of bioactivity or biodegradation by manipulating the HA/*β*-TCP ratio [5]. Concentrated studies on their development and applications were made by Daculsi et al. [6–8].

Thus, through the combination of a balanced rate between a more stable phase (HA) and a more soluble one (*β*-TCP), it was possible to formulate a BCP with a controlled dissolution rate and different mechanical properties [5]. The presence of porosity and a bioactive surface facilitate cell attachment, proliferation, and differentiation and, consequently, provide a more biocompatible, osteoconductive, and in some cases, osteoinductive ceramics, which can favor increased bone formation [6, 7, 9–20]. Indeed these chemical and physical properties, produced by variations of the temperature, pH and duration of the sintering process, make each biomaterial unique and lead to different tissue responses [7, 10, 12, 13, 16–18]. Currently the BCP represents the most promising and best alternative for bone reconstructions as they can overcome the shortcomings of the autografts and allografts such as expense, limited supply, additional trauma in the case of autografts, and risk of disease transmission in the case of allografts.

The objective of this study is to provide a retrospective evaluation, through the analysis of randomly selected clinical cases, of the long-term efficacy and performance of BCP bioceramics as bone substitutes for the reconstruction of defects with different morphologies and caused by distinct etiologies.

## 2. Methods

### 2.1. Biphasic Calcium Phosphate (BCP) Bioceramics

The BCP bioceramics (Osteosynt^®^, EINCO Biomaterial Ltda., Belo Horizonte, Minas Gerais, Brazil) used for the reconstruction of the defects in 66 patients is composed of 65% of HA and 35% of *β*-TCP, with a tolerance of ±5%, and characterized by the presence of intercommunicating micro- and macroporosity of <10 *μ*m and >100 *μ*m, respectively. The two different forms used were the granular form (20–40 mesh and 40–60 mesh) and a prefabricated wedge. The characterization of the crystalline phases of the BCP was performed using X-ray diffraction (XRD) (Phillips-PANalytical PW1710, USA); the presence and sizes of porous were demonstrated by scanning electron microscopy (SEM) (S-3500N, Hitachi).

### 2.2. Clinical Cases

This study was approved by the Ethics Committees of the Hospital São Bento Cardioclinica S.A. (Belo Horizonte, Minas Gerais, Brazil) and all the subjects participating in the study signed a consent form.

This study is a retrospective evaluation of 66 randomly chosen clinical cases of patients who had undergone orthopaedic surgeries with BCP as bone graft substitute.

These sixty-six patients corresponded to 67 surgical procedures (one patient had bilateral interventions). Thirty seven patients were males and 29 females. One patient was 1-year old and one, 70-year old. The ages of the other 64 patients ranged between 15-year and 69-year old with a mean age corresponding to 41.6 years (Table 1). 

The defects were classified according to the etiology of the lesions and the morphology of the bone defect. Regarding the etiology, the defects were divided into the following: (1) orthopaedics defects (32 patients and 33 defects that corresponded to 33 surgical procedures), comprised of bone defects resulting from elective surgical procedures, such as total hip prosthesis (placement and replacement), osteotomies, and arthrodesis and (2) traumatic defects (34 defects corresponding to 34 surgical procedures), which included noninfected pseudoarthrosis, fractures, and arthrodesis after posttraumatic arthrosis.

Regarding the morphology, the defects were divided into: (1) cavitary bone defects (42 defects) and (2) segmental bone defects (or gap defects) (25 defects). The volume of the cavitary bone defects were not measured due to the irregularity of their shape. The size of segmental bone defects was measured from the X-Ray images, and their largest length was noted.

### 2.3. Surgical Procedures

The surgical technique used for the implantation of BCP followed the surgical principles that are equivalent to those required for autografts: curettage and debridation of the wound site until viable bone tissue could be observed and impaction of the bioceramics. The bioceramics was applied directly into the bone defects and no extra volume of the bioceramics was used except for the arthrodesis procedures where the creation of an external bridge between the bone extremities was necessary. External or internal fixation systems (AO/ASIF methods) were used according to the indications for each patient.

In acute traumatic lesions, open reductions were performed, and the defects were immediately filled with the BCP.

Three patients received a combination of autografts and BCP: one presented a pseudoarthrosis of the femur with a gap bone loss of 11 cm in length, one had a bimalleolar fracture of the ankle (considered a cavitary bone defect), and the other presented a pseudoarthrosis of the tibia, with a bone gap of 4 cm in length.

### 2.4. Clinical and Radiographic Evaluation

The classification of the final results was based on clinical and radiographic assessments. For the clinical evaluation, the functional movements of the operated limb, without pain, at the end of the treatment, were measured, and scores ranging from 0 to 3 were given as follows:

score 0: absence of movement of the operated arch or limb,
score 1: increased limitation of movements,
score 2: same range of movement compared to the preoperative analysis, and
score 3: normal range of motion of the operated arch or limb.

For the radiographic analysis, the presence or absence of integration and the number of surgical procedures performed until the final outcome were considered. The following scores were given:

score 0: no integration of the bioceramics, independent of the number of surgical procedures;
score 1: more than one surgical procedure was performed in order to observe the bioceramics integration (and bone formation);
score 2: the bioceramics were integrated and the patient healed after the first surgical procedure.

The final results represent the combination of both parameters, clinical + radiographic scores: 0 to 2 (poor results), 3 (moderate results), 4 (good results), and 5 (excellent results).

## 3. Results

**Figure 1:**
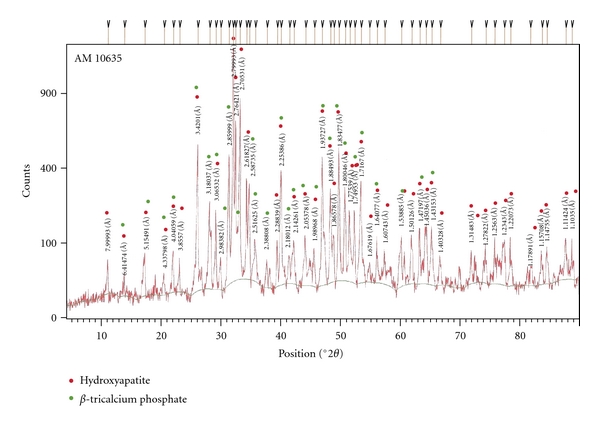
X-Ray diffraction of bioceramics. This analysis shows the crystalline phases of the BCP and the HA/ß-TCP ratio which corresponds to 65%/35%, respectively.

**Figure 2:**
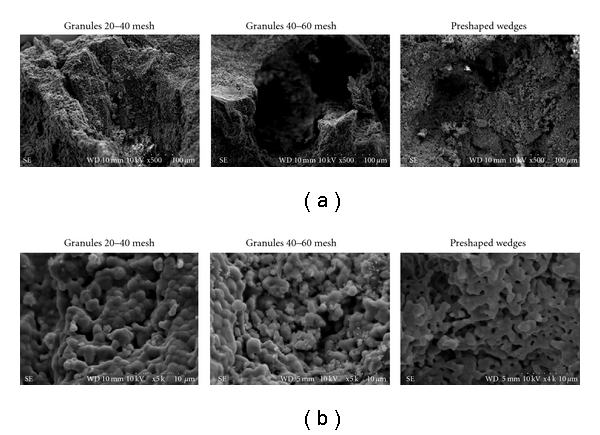
SEM images showing the physical structures of the granules 20–40 mesh, 40–60 mesh and preshaped wedge. The macroporosities (a) and microporosities (b) can be observed in all the samples.

The XRD diffraction analysis demonstrated the HA/ß-TCP ratio of the BCP (Figure 1), and the SEM images showed the presence of micro- and macroporosities in the three presentation forms of the bioceramics used in this study (Figure 2). 

Of the 67 procedures performed, five were classified as excellent results (combined score of 5), fifty seven procedures were classified as good (combined score of 4), and five were classified as moderate (combined score of 3). 

The defects were classified according to the etiology of the bone loss, that is, orthopedic or traumatic defects, and the morphology of the bone defect, that is, segmental (also called gap defects), or cavitary defects, such as cystic lesions.

 In the group classified according to the etiology of the defects, thirty two patients underwent thirty three procedures for bone reconstruction with BCP. These patients had orthopedic bone defects associated with hip or knee prosthesis placement or revisions, osteotomies, and arthrodesis. In this group, complete integration of the BCP after the first surgical procedure was observed and patients experienced improved or the same range of motion of the operated limb or arch without pain. Thus, thirty procedures were classified as good results (score 4) and three had excellent results (score 5) (Table 2). 

Thirty four patients underwent 34 surgical procedures to treat traumatic defects. Two of them had excellent final results (score 5), twenty seven had good final results (score 4), and five were classified as moderate results (score 3). Four out of the five moderate results required an additional surgery to achieve maximum bone formation in the operated site, and one patient had an increased limitation of the limb movements after the treatment (Table 2). 

Regarding the morphology of the bone defect, 24 patients with segmental defects underwent 25 procedures, and 42 patients with cavitary bone defects required 42 procedures. Results of the BCP in repairing cavitary bone defects showed good results for 40 procedures (score 4) and excellent results for 2 procedures (score 5) (Table 3).

Nineteen procedures were performed in segmental bone defects smaller than 3 cm in length and 6 procedures were performed in 3 cm defects or larger (Table 4). 

**Figure 3:**
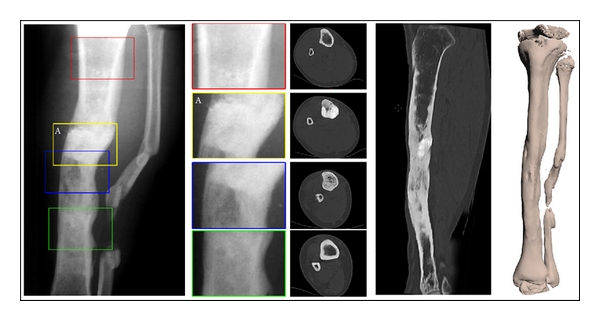
Bone lengthening in an area previously reconstructed with BCP. X-Ray and CT images of a tibia showing the zone reconstructed with BCP (A) and the new bone formed after osteotomy and bone lengthening, which was performed due to the shortening of the limb.

Within the segmental defects that were smaller than 3 cm in length, three of them presented excellent results (score 5), 15 were classified as good results (score 4) and one had a moderate result (score 3). It is important to highlight, however, that in this particular case, the moderate result was not due to problems with the bioceramics but due to the shortening of the limb at the end of the treatment. As a consequence, the patient required a second surgical procedure, where the osteotomy for the limb lengthening was performed throughout the area that had been previously reconstructed with the bioceramics (Figure 3). 

Of the 6 patients with bone defects equal or bigger than 3 cm in length, only two of them showed healing after the first surgery. One of them, however, received score 3 (moderate result) instead of 4 (good results) due to increased limitation of the knee movements. Three patients required a second surgical intervention, and one patient, with a defect of 11 cm in length, required a third procedure. Consequently, 2 procedures were classified as good (score 4) and 4 procedures, as moderate (score 3).

Only three patients out of 66 received BCP combined with autologous bone grafts in the first surgical intervention. These results were different for each of these 3. One patient had a cavitary bone defect that healed well, receiving score 5, one patient, with a segmental bone defect of 4 cm in length, had a nonunion and required an additional surgical procedure using only BCP without autografts and received score 3, and one patient with 11 cm length segmental defect required an additional surgery, receiving score 3 at the end of the treatment (Table 4).

None of the patients experienced poor results, that is, no integration of the bioceramics (Table 5) that could be either associated or not to an increased limitation or absence of movements.

## 4. Discussion

This study evaluated the efficacy of a biphasic calcium phosphate (BCP) bioceramics in the reconstruction of bone defects caused by trauma or associated with elective procedures such as prosthesis placement and revisions, arthrodesis, and osteotomies (called orthopedic defects) as well as in the reconstruction of cavitary and segmental bone defects.

**Figure 4:**
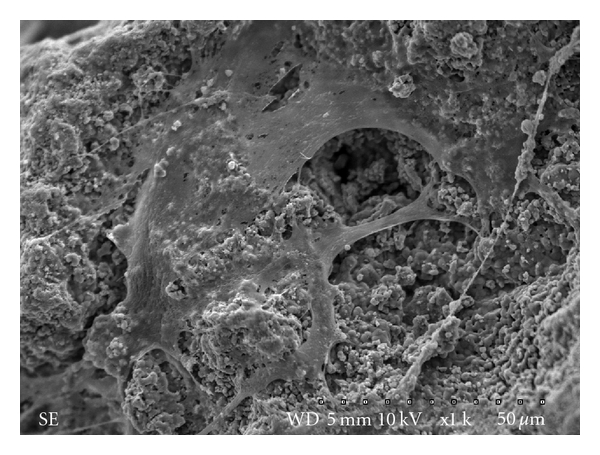
Human mesenchymal stem cells on the surface of a granule (40–60 mesh). SEM image shows stem cells spreading on the BCP surface and forming layers over the apertures of porous, after 7 days of culture.

A scale of different scores was used in order to measure the clinical and radiographic outcomes. Similar scoring scale has been used in a previous study [21]. We have shown that the BCP was successful in regenerating bone in all the cavitary and in segmental bone defects where the gaps were smaller than 3 centimetres in length. Most of these defects were also classified as orthopaedic defects. Segmental defects equal or larger than 3 centimetres in length, which were mostly caused by trauma, required special attention and eventually needed an additional surgical procedure to heal. In some of these patients, as the healing period was longer than for those with smaller defects, the substitution of the pins and nails used for fixation was required due to their movements and instability over time. None of the patients presented acute inflammatory reactions or infections. One of the factors that has been described as eliciting response of cells such as monocytes, which are among the first cells to contact the implant surface and to colonize the inflammatory site, is the ratio between the surface area of cells and the surface area of the biomaterial [22, 23]. The ratio related to the surface area of cell/surface area of material equivalent to one has been shown to elicit the highest level of inflammatory cytokine production [22, 23]. The granule sizes used in these clinical cases are much bigger than all cells, as shown by the SEM analysis (Figure 4). The chemical composition, topography, wettability, and surface energy of the biomaterial are other factors that will influence cell response [24, 25]. 

**Figure 5:**
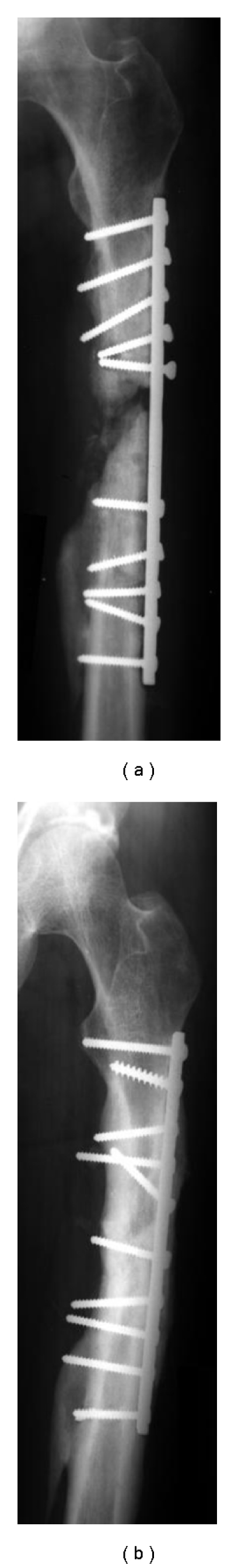
Reconstruction of a femoral bone defect with BCP. X-Ray images of a femur before (a) and after (b) reconstruction with the BCP and stabilized with internal fixation system, after trauma.

**Figure 6:**
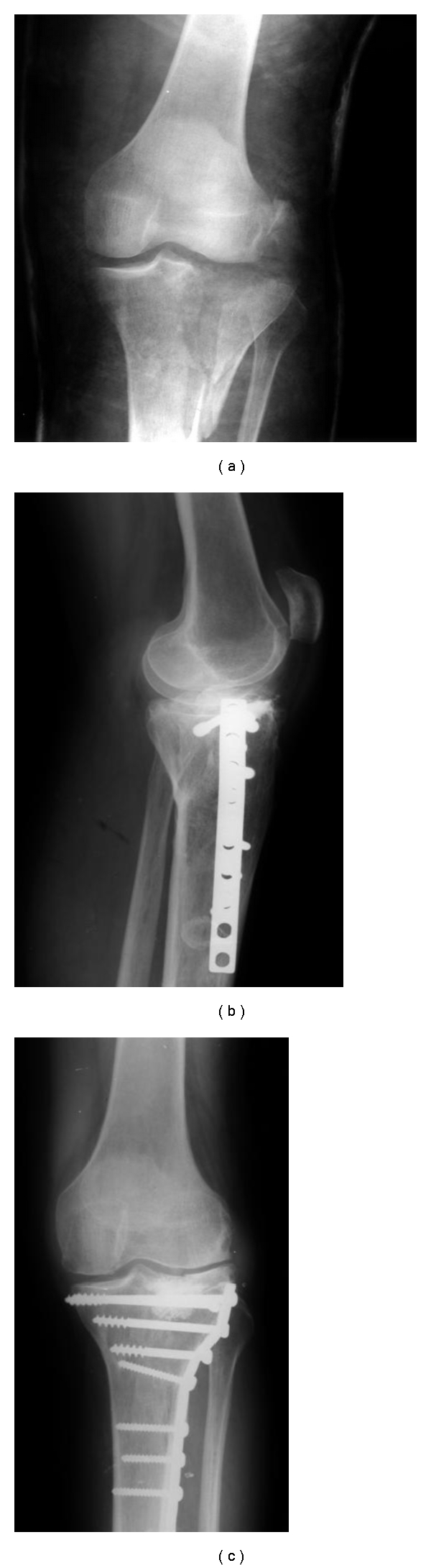
A tibial bone defect reconstructed with BCP. X-Ray image of a tibial fracture (a) with the lateral (b) and anterior view (c) after 2 years of reconstruction using the BCP.

Our radiographic analysis showed a higher radiopacity at the implant site even in the long-term evaluation (Figures 3, 5, and 6). This is in agreement with the study reported by Rouvillain et al. [26] that showed the same image characteristic even after 18 months of the implantation of BCP (60% HA/40%  *β*-TCP) wedges in high tibial valgisation osteotomies. These authors gave evidences of a resorption rate corresponding to more than 60% of the bioceramics after 2 years and demonstrated that the high radiopacity was due to the greater mineral concentration and the composite formed by the new bone tissue and the residual granules and not due to the nonresorption of the biomaterial [26]. Our good results are in agreement with those reported by others [27].

Since the first successful clinical application of commercial calcium phosphate bioceramics in the early 1970s, modifications on their properties gave rise to alternatives with improved biological and mechanical properties [7, 9, 10, 17, 28–30].

The BCP used in this study consisted of 65% of HA and 35% of *β*-TCP and belongs to the third generation of biomaterials, namely, those having appropriate micro- and macroporosities, good mechanical properties, and promoting not only bone substitution but also bone regeneration [9]. An animal study showed that such bioceramics, in the granular form (40–60 mesh) promoted a bone mass gain up to 4-fold the initial volume used [20]. 

The nature, timing and progression of the bone regeneration, was shown to be influenced not only by the bone physiology, biomechanical properties at the site of the lesion, age, and intrinsic conditions of the patient but also by the chemical composition and physical structure of bone graft substitute, which will directly influence the migration and proliferation of the host cells [7, 10, 15, 18, 19, 31–33].

For a biomaterial to function as a good scaffold, it must not be prematurely resorbed before the new bone formation occurs. For this reason, highly soluble compounds, such as calcium sulphate, calcium carbonate, dicalcium phosphate dihydrate, octacalcium phosphate, and beta-tricalcium phosphate alone, may not be suitable materials for the reconstruction of major bone defects in humans, even if *in vitro* cell response to these calcium phosphate materials is positive. Bioceramics with rapid resorption or dissolution rate promotes an important change of the local pH due to the high levels of calcium ions released, leading to a mild inflammatory process and/or to a fibrous encapsulation of the material [31, 34] resulting in an unsuccessful bone regeneration.

Thus, in the time-dependent process of bone formation, the degradation rate is a key point for the clinical success, mainly when larger defects are being treated. Indubitably the use of BCP with different ratios of low soluble component (HA) and a highly soluble phase (*β*-TCP) is an option to tailor the degradation kinetics of calcium bioceramics from a few weeks to a few years [5, 7, 10, 11, 15].

Some calcium phosphate (CaP) bioceramics with different physical structures (including the size and density of porosity) and chemical composition have been used alone or mixed with adjuvants of bone healing (e.g., fibrin sealant, platelet-rich plasma and stem cells) in several clinical situations with good results [35–43]. However, in most of these cases, the bioceramics were applied in relatively small bone defects. 

Different results were obtained from the 3 patients who were treated with a combination of BCP/autografts. Considering that this number of patients is very low and the results are not consistent, no conclusion can be made about the possible benefits of combining autografts with BCP. Further studies still must be performed in order to collect evidences that support the real advantages of such strategy.

Another crucial point that must be taken into account for a successful clinical treatment is the correct preparation of the surgical site. All the surgical technique principles that are indicated for the autologous bone grafting must be applied for the bioceramics implantation. Indeed, avoiding infected areas, eliminating all the granular tissues and fibrosis at the surgical site, and impacting the biomaterial where healthy and bloody bone extremities are observed are crucial steps.

## 5. Conclusions

This study showed clinical evidences of the efficacy of the micro- and macroporous biphasic calcium phosphate (BCP) bioceramics with 65HA/35*β*-TCP ratio, in the reconstruction of not only small but also large bone defects in humans. Results from this study suggest that such material can be used in load-bearing areas and represents a safe and predictable alternative for autografts and allografts. In case of segmental bone defects equal or larger than 3 cm in length, the association of BCP scaffolds, such as the ones used in this study, with bioactive molecules or stem cells may be considered. 

Knowledge of the bioceramics properties and application of appropriate surgical techniques are critical aspects for good clinical outcomes.

## Figures and Tables

**Table 1:** Description of age, gender, and affected bone of each patient. The initial diagnosis is described in parenthesis. F: female; M: male.

Procedure ID	Age	Gender	Bone
01	25	F	Femur (pseudarthrosis)
02	50	M	Hip prosthesis (revision)
03	51	F	Femur (pseudarthrosis)
04	32	M	Knee (osteoarthrosis)
05	51	F	Tarsus (osteoarthrosis)
06	69	F	Ankle (pseudarthrosis)
07	53	M	Hip prosthesis (revision)
08	64	F	Hip prosthesis (revision)
09	33	M	Tibia (fracture)
10	49	M	Femur (osteonecrosis)
11	46	M	Calcaneus (fracture)
12	20	M	Femur (osteonecrosis)
13	53	M	Femur (pseudarthrosis)
14	34	M	Radius (pseudarthrosis)
15	46	M	Femur (osteonecrosis)
16	37	M	Ankle (fracture)
17	38	F	Tibia (pseudarthrosis)
18	41	F	Radius (fracture)
19	38	F	Acetabulum (reconstruction)
20	16	F	Tibia vara
21	35	F	Calcaneus (fracture)
22	43	F	Clavicle (pseudarthrosis)
23	28	M	Carpal bone (arthrosis)
24	42	M	Hip prosthesis (revision)
25	58	M	Hip prosthesis (revision)
26	27	M	Femur (pseudarthrosis)
27	01	M	Clavicle (congenital pseudarthrosis)
28	65	F	Hip prosthesis (revision)
29	50	M	Hip prosthesis (revision)
30	60	F	Hip osteoarthrosis
31	36	M	Humerus (fracture)
32	70	M	Wrist (osteoarthrosis)
33	24	M	Radius (fracture)
34	33	M	Calcaneus (fracture)
35	32	M	Forearm (pseudoarthrosis)
36	28	M	Forearm (pseudarthrosis)
37	46	F	Femur (pseudoarthrosis)
38	56	M	Ankle (fracture)
39	41	F	Knee (osteoarthrosis)
40	44	F	Femur (osteotomy)
41	33	M	Femur (fracture)
42	49	F	Ankle (fracture)
43	22	F	Femur (pseudoarthrosis)
44	34	F	Calcaneus (fracture)
45	01	M	Clavicle (congenital pseudarthrosis)
46	60	F	Humerus (pseudarthrosis)
47	30	M	Tibia (pseudarthrosis)
48	59	M	Tibia (pseudarthrosis)
49	56	M	Radius (fracture)
50	23	F	Humerus (fracture)
51	30	M	Humerus (pseudarthrosis)
52	34	F	Ankle (arthrosis)
53	57	F	Elbow (osteoarthrosis)
54	33	M	Femur (fracture)
55	59	F	Humerus (pseudarthrosis)
56	40	M	Femur (osteonecrosis)
57	15	F	Femur (genu valgum)
58	37	M	Tibia (fracture)
59	64	F	Hip prosthesis (revision)
60	63	M	Tibia (fracture)
61	21	F	Femur (genu valgum)
62	19	M	Femur (pseudoarthrosis)
63	46	M	Femur (pseudoarthrosis)
64	38	M	Knee (osteoarthrosis)
65	50	F	Hip prosthesis (revision)
66	62	F	Hip prosthesis (revision)
67	38	M	Ankle (pseudoarthrosis)

**Table 2:** Final results according to the etiology of the bone defects. The number of patients and procedures and the final scores given to patients with orthopaedic and traumatic defects are described. All procedures performed in orthopaedic defects presented good or excellent results. Defects caused by trauma are more suitable to show moderate results.

Etiology of the defect	Number of patients	Total number of procedures	Number of procedures with excellent results (score 5)	Number of procedures with good results (score 4)	Number of procedures with moderate results (score 3)
Orthopedic defects	32	33	3	30	0
Traumatic defects	34	34	2	27	5

Total	66	67	5	57	5

**Table 3:** Final results according to the morphology of the bone defects. According to the morphology, most of the defects were classified as cavitary bone loss and presented good results. Moderate results were observed mainly in segmental defects equal or bigger than 3 cm in length.

Morphology of the defect	Number of patients	Total number of procedures	Lengths of the defects	Number of procedures with excellent results (score 5)	Number of procedures with good results (score 4)	Number of procedures with moderate results (score 3)
Segmental defects	24	25	<3 cm = 19 ≥3 cm = 6	<3 cm = 3 ≥3 cm = 0	<3 cm = 15 ≥3 cm = 2	<3 cm = 1 ≥3 cm = 4
Cavitary defects	42	42	Not measured	2	40	0

Total	66	67	—	5	57	5

**Table 4:** Lengths of the segmental bone defects. Nineteen procedures were performed in defects smaller than 3 cm in length and six, in defects equal or larger than 3 cm. The final scores (combination of clinical and radiographic analysis) are described. One patient received bilateral intervention. The majority of good and excellent results (scores 4 and 5) were observed in patients with defects equal or smaller than 3 cm in length.

Procedure ID	Length of the defect	Bone affected	Final score
1	11	Femur	3
3	2	Femur	4
13	3	Femur	3
14	1.5	Femur	4
17	1	Tibia	4
22	1	Clavicle	5
26	3.5	Femur	4
27	0.5	Clavicle	5
35	2.5	Forearm	4
36	1	Forearm	4
37	0.5	Femur	4
40	1	Femur	4
41	5	Femur	3
43	1	Femur	4
45	0.5	Clavicle	5
46	1.5	Humerus	4
47	2	Tibia	3
48	4	Tibia	3
49	1.5	Radius	4
50	2.5	Humerus	4
51	3	Humerus	4
54	2	Femur	4
55	2.5	Humerus	4
62	2	Femur	4
63	1	Femur	4

**Table 5:** Results of the radiographic analysis according to the etiology of the bone defects. Most of the patients received score 2, corresponding to the integration of the bioceramics after the first surgical procedure.

Etiology of the bone defects	Number of procedures according to the radiographic scores		
	Score 0	Score 1	Score 2
Traumatic defects	—	4	30
Orthopedic defects	—	—	33
